# Patterns of Seed Penetration by the Date Stone Beetle *Coccotrypes dactyliperda* (Coleoptera, Curculionidae, Scolytinae)

**DOI:** 10.3390/insects13010010

**Published:** 2021-12-22

**Authors:** Dirk H. R. Spennemann

**Affiliations:** Institute for Land, Water and Society, Charles Sturt University, P.O. Box 789, Albury, NSW 2640, Australia; dspennemann@csu.edu.au

**Keywords:** spermatophagy, feeding behavior, locomotion, activity patterns

## Abstract

**Simple Summary:**

Date stone beetles tunnel into the seeds of several palm species, where they establish brood galleries in which they spend their entire life cycle. Their larvae, which consume the seed’s albumen, pupate and hatch in the seed, with multiple generations co-existing until all the seed’s albumen has been consumed. Little is known about the initial tunneling and nature of the establishment of the brood galleries. Through experimentation with seeds of the Canary Islands Date Palm, this study shows that the beetles exhibited an overwhelming preference for a penetration at the grooved side. Behavioral observations showed that, in order to penetrate, the date stone beetle needs to be able to push its mandibles into the epicarp of the seed. The main thrust is generated by the hind legs which requires traction as provided by the groove. When tunneling, the mid legs function as a pivot, while the fore legs enable lateral control. When in the tunnel cavity the pair of mid legs act as wall anchors. Gustatory cues prevent the beetle from tunneling through to the other side. The nature of these gustatory cues remains unclear at this point and awaits future research.

**Abstract:**

The cryptic spermatophagus date stone beetle (*Coccotrypes dactyliperda* Fabricius, 1801) tunnels into palm seeds for oviposition and subsequent establishment of brood galleries. Based on behavioral observations this paper describes the biomechanical and gustatory parameters that govern the initial excavation of the tunnels and the subsequent establishment of the galleries. When tunneling into Canary Islands Date Palm (*Phoenix canariensis* Chabaud, 1882) seeds, penetration principally occurs at the dorsal side of the seed, in particular the groove, which allows the beetle to gain the required traction. Tunneling is executed in a circular fashion with clockwise or counter-clockwise repositioning in approximately one-eighth to one-quarter turns. Biomechanically, the three pairs of legs provide thrust (hind legs), pivoting (mid legs), and lateral control (fore legs). Gustatory cues, the nature of which remains unclear at this point, prevent the beetle from tunneling through to the other side.

## 1. Introduction

The date stone beetle, *Coccotrypes dactyliperda* (Fabricius, 1801), is a cryptic spermatophagus beetle of the Curculionidae family (Coleoptera: Curculionidae: Scolytinae: Dryocoetini), with females measuring 1.9 to 2.2 mm in length and about 0.7 to 1 mm in width. The beetle, which has a convex appearance and is hairy across the dorsal surface, ranges in color from reddish brown to almost black brown [1,2,3,4].

As with other crypto-parasites, the entire life cycle of *C. dactyliperda* occurs inside the seed [5,6,7,8,9,10]. After emergence from hibernation [11,12], the first generation of female beetles to leave the brood chamber (‘gallery’) emerges during late December/early January in the southern hemisphere (late July/early August in the northern hemisphere) and attacks the green drupes of the date palm (*Phoenix dactylifera* L.), causing the bulk of these to abscise one to two days later [13,14]. The species also predates the seeds of fallen dates, often after the pericarp has been consumed by other animals, such as rodents. This continues until August, when a second generation emerges from the seeds. The rate of abscission varies, but when the infructescences are not protected from beetle attack by chemical or physical (bags) means, production losses usually range between 20 and 40% [15,16,17]. *C. dactyliperda* also attack other palms, in particular the Canary Island date palm (*Phoenix canariensis* Chabaud, 1882) [18,19].

Initially distributed in the Middle East and North Africa as part of the date palm horticultural complex, the distribution range of the species has seen a remarkable increase during the nineteenth century, mainly due to the trade in dates as fruit for human consumption, the distribution of palm seeds (in particular *P. canariensis)* for horticultural endeavours, and in the form of vegetable ivory (*Phytelephas* spp. palm) for button manufacture [20,21,22]. Today, *C. dactyliperda* has become a true cosmopolitan species that can be found in most subtropical and temperate zones [23].

**Figure 1:**
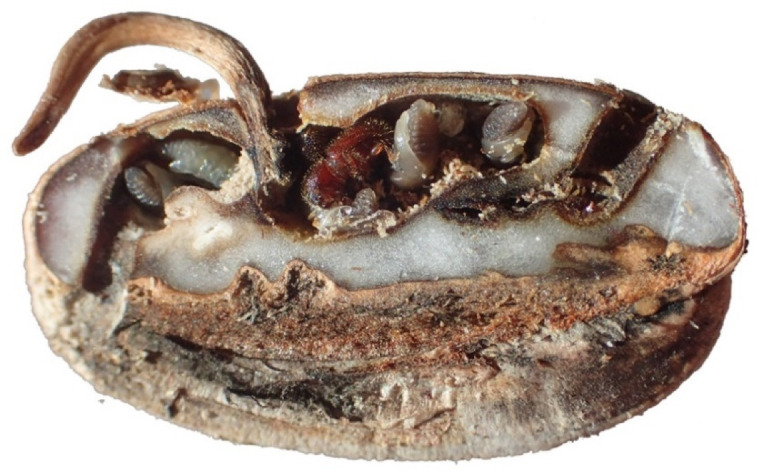
A *Coccotrypes dactyliperda* gallery impacting on the cotyledonary petiole of a *Phoenix canariensis* seed. The seed was split lengthwise through the dorsal groove. Ventral side is on top, dorsal side on bottom of image.

Germination experiments showed that while the majority of the seeds damaged by *C. dactyliperda* failed to germinate, some of the seeds germinated, despite penetration [24]. The duration of the growth of the cotyledonary petiole (often mistakenly called the radicle) varied, depending on the extent of the brood gallery (Figure 1). This suggests that the location of the initial penetration, and in particular the location of the gallery, may be of significance. Seeds where the embryo was affected by initial tunneling failed to germinate, while the others did. The progressive consumption of the albumen (endosperm) by the beetle, however, implies that the emerging cotyledonary petiole has less energy at its disposal and is therefore more likely to wilt before it can form viable rootlets and a photosynthetically active leaflet.

As this has direct implications on the recruitment success of palms, it was therefore of interest to examine whether there are consistent patterns in the way *C. dactyliperda* tunneled into *P. canariensis* seeds. To date, none of this has been examined, primarily due to the cryptic nature of the beetle species.

During the breeding of quantities of *C. dactyliperda* specimens for use in a containment experiment [25], it was noted that the beetles appeared to predominately penetrate the dorsal side of the *P. canariensis* seed which exhibits the central groove.

Using experimental and observational data, this paper will examine the placement and nature of tunneling in order to understand whether there are consistent discernible patterns in the way *C. dactyliperda* tunnel into *P. canariensis* seeds.

## 2. Methods

The *Coccotrypes dactyliperda* beetles used here were obtained from a population at the PC2 laboratory of the Peter Till Laboratories, Faculty of Science, Charles Sturt University (Albury, Australia), reared for use in a multi-factorial experiment, assessing food choices and emergence times [26]. All experiments were conducted between October 2018 and March 2019. The original beetle population stemmed from *Phoenix canariensis* seeds collected at Alma Park, NSW, Australia [26].

### 2.1. Designs

There are three principal experimental designs: one based on data collected during the preparation of samples for experiments for other purposes (Section 2.1.1), a retrospective examination of experiments (for other purposes) already under way (Section 2.1.2), and one experiment specifically designed for this paper (Section 2.1.3).

#### 2.1.1. Mass Exposure Experiments

Observations for mass exposure experiments could be obtained when beetles were reared for a range of containment and resilience experiments, all of which required seeds in which *C, dactyliperda* beetles had commenced to breed [25,27,28]. The nature of these experiments had no bearing on this study. Available are data from seven replicates where 30 beetles were exposed to 30 freshly peeled seeds (placed in 200 mL sample jars) (sample #1 (Table 1; Figure S1). Some of these mass exposure events resulted in multiple penetrations of individual seeds. These were excluded from the primary analysis, but these multiple penetrations are detailed in Table 4. The resulting actual sample sizes range from 13 to 29 with a pooled total of 134.

Another experiment required fifteen replicates of 10 beetles each [27]. Five of the mass exposure events resulted in multiple penetrations of individual seeds. These replicates were excluded from analysis. The remaining ten sets form experiment #2.

#### 2.1.2. Retrospective Examination

Existing samples used in experiments which were running at the time were retrospectively examined for the location of penetration: seeds used in a germination experiment set out in 9 × 18 grids in plastic tubs (on a seed bed) (sample #3) and seeds used in a germination experiment set out in 4 × 5 grids in plastic bags (lined with moist kitchen tissue) (sample #4). The original population of beetles had emerged from seeds that were not known to be infested and which had been unintentionally included in the germination experiments (samples #3 and #4) [24]. During the retrospective examination of these samples, the older seeds from which the initial beetle population had emerged were readily recognizable by multiple exit holes and were omitted from the recording. Because the breeding in samples #3 and #4 occurred uncontrolled, the starting number of beetles is not accurately known.

The third retrospective sample comprised single seeds exposed to single beetles as part of a food choices experiment (sample #5) [26].

#### 2.1.3. Exposure to Seeds with Open Operculum

Following the observation of a distinct preference of location, it was of interest to assess whether this might hold true, even when beetles had the opportunity of accessing an existing opening or point of access. Selected were *P. canariensis* seeds that had originally been used in a germination experiment [24] and which had germinated (emergence of cotyledonary petiole) but which showed no penetration. The desiccated cotyledonary petiole was scraped off at the operculum, leaving a 1.5 to 2 mm wide opening with a shallow, 0.1 to 0.5 mm deep depression. To account for differences in the age of seeds, three samples were prepared, each comprised of 30 beetles exposed to 30 seeds: one set of (originally) freshly peeled seeds (sample #6), one set of light brown seeds (2018 season, sample #7), and one set of black seeds (2017 season, sample #8) [24]. As before, seeds with multiple penetrations of individual seeds were excluded (but see Table 4).

#### 2.1.4. Nature of Penetration

During the assessment of food choices [26], individual *C. dactyliperda* specimens were exposed to food options in clear 200 mL plastic sample jars (Figure S1), where they could be observed exploring and tunneling into seeds. Four seeds where tunneling was in process were extracted from the jars and placed in front of a camera to video record the tunneling action. These were augmented by observations of *C. dactyliperda* specimens exposed to seeds from the Mexican Fan Palm (*Washingtonia robusta*) and hazelnut (*Corylus avellana*) as well as a blank for a Tagua button (*Phytelephas* spp. palm).

### 2.2. Recording

#### 2.2.1. Location of Penetration

**Figure 2:**
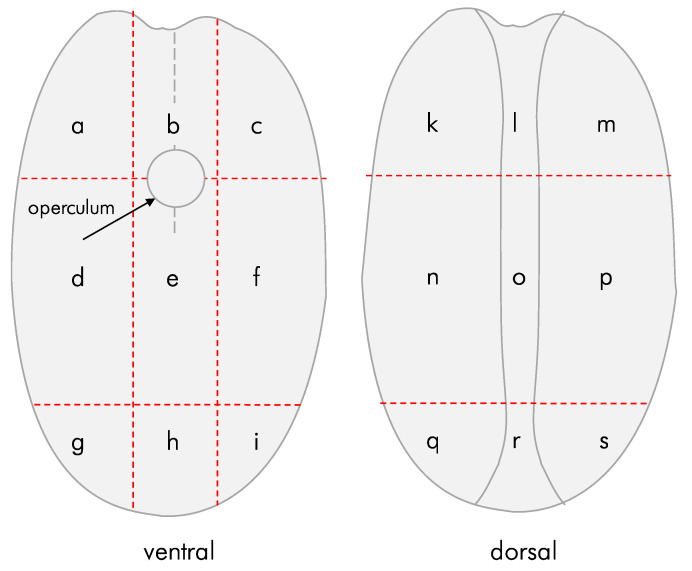
Classification of the location of the initial entry hole. Letters consecutively identify arbitrarily defined sectors on *Phoenix canariensis* seeds.

To record the positioning of the penetration hole, the curved front (ventral) and flattish back (dorsal) of a seed were divided into nine sectors each (Figure 2). The partitioning into the sectors was based on morphological criteria of *P. canariensis* seed to ensure ready and replicable recording. The positioning of the penetration hole was recorded (Figures S2–S9). The penetration was observed and recorded within 48 h after initial exposure, which ensured that only penetration and not exit holes were recorded. While the sectors are symmetrical on the ventral and dorsal side, their surface area differs. All sectors, with the exception of sectors d, f, n, and p can be considered to have a similar surface area (with the groove sectors l, o, and r having vertical areas not shown on Figure 2). Sectors d, f, n, and p have approximately 1.5 times the area of the other sectors.

#### 2.2.2. Nature of Penetration

Four seeds where tunneling was in process were placed in front of a camera (Olympus TG-3 camera with ‘microscope’ macro) and the tunneling efforts video recorded (Figures S12–S20). The footage was viewed and the activity of the beetle recorded. The activities of gnawing (G), debris removal (DR), and pauses (P) were coded. Movement was recorded as clockwise (CW) and counter-clockwise (CCW). The direction of the camera was coded as ‘N’ and the orientation of the beetle’s pronotum in relation to the camera direction expressed as octants of the cardinal directions (i.e., N, NE, E, SE, etc.). The footage was shot either hand-held or tripod mounted.

For the sub-zero temperature experiment, beetles had been allowed to tunnel for 49 h before a number of *P. canariensis* seeds were exposed sub-zero temperatures [27]. All seeds used were cut open to examine whether breeding continued after exposure [27]. Seeds that were subjected to prolonged exposure to cold temperature, show the early stages of tunneling at the time of oviposition as the beetles perished.

### 2.3. Statitics

The statistical analysis tested three null hypotheses: (i) that there would be no difference in penetration rates between the sectors as defined in Figure 2, (ii) that any observed differences would not differ from a set of random generated locations, and (iii) that there would be no difference between smooth and rough surfaces.

For the purpose of inter-sector analysis, the area difference between sectors d, f, n, and p and the other sectors was ignored in the first instance and was only applied if differences were not significant at the initial stage.

The random distribution of penetration holes was obtained using the RAND function in MS Excel.

The significance assessment of observed differences in frequencies and percentages used the Chi-squared test with *n* − 1 correction of the MEDCALC comparison of proportions calculator [29,30,31].

## 3. Results

The observational and experimental results will be discussed by first considering the location of penetration, to be followed by observations of beetles tunneling into seeds. Additional tables and images are provided in a supplementary data file. These are indicated by the prefix ‘S’.

### 3.1. Location of Penetration

The data show an overwhelming preference for a penetration at the dorsal side of the seed, with locations in the groove (l, o, r) more common than others (Table 2). Penetration of the ventral side was uncommon and occurred in greater numbers only in the case of seeds that had germinated and where the operculum was open, thereby providing access to the pericarp (samples #6–#8).

Where opercula could be accessed (samples #6–#8) all penetration on the ventral side ceased and even the percentage of dorsal perforations decreased (Table 3). There was little difference in the location of perforations between samples of single seeds (samples #3, #5) and bulk seeds (samples #1, #8). Only where seeds were placed between sheets of tissue (for germination) did the percentage of perforations on the ventral side increase (sample #4). Setting aside that sample, the only location on the ventral side that showed penetration was sector ‘e’, where the ventral side of a seed is at its flattest.

Sample #4 shows a much greater variation in the positioning of penetration, even though the groove is still the preferred location (*p* < 0.01, Table 2). Significantly, in this sample the combined percentage of penetrations at the ventral surface is the highest (19.8%) of all samples, except for those with open opercula. Sample #4 differed from the others inasmuch as the seeds had been placed between sheets of moistened kitchen tissue (to facilitate germination). Clearly, the sheets provided the beetles with traction that allowed them to penetrate the smooth sides.

As noted in the methodology, experiments #1 and #2 were set up with replicates. Location ‘l’ was significantly preferred in all replicates in experiment #1 (Table S1) and in 60% of the replicates of experiment #2 (Table S2).

Based on the confidence limits of the proportions set out in Table 3, the penetration of the seeds occurs in all cases significantly more frequently (*p* < 0.001) in the dorsal groove than the ventral side (samples #1–#5) or the operculum (samples #6–#8) [32].

**Figure 3:**
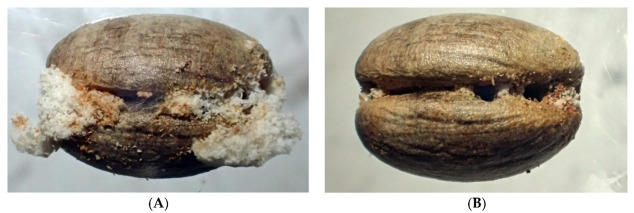
Triple penetration of a *Phoenix canariensis* seed. (**A**) With frass; (**B**) cleaned.

**Figure 4:**
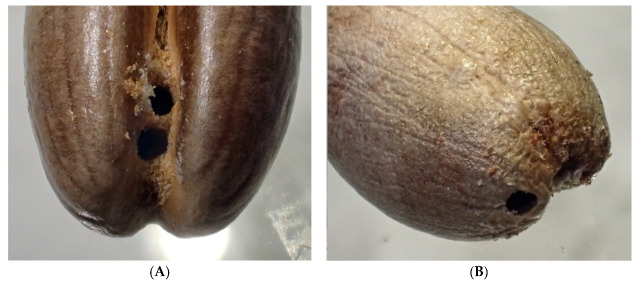
Triple penetration of a *Phoenix canariensis* seed. Details of Figure 3. (**A**) Dorsal view; (**B**) ventral view.

**Figure 5:**
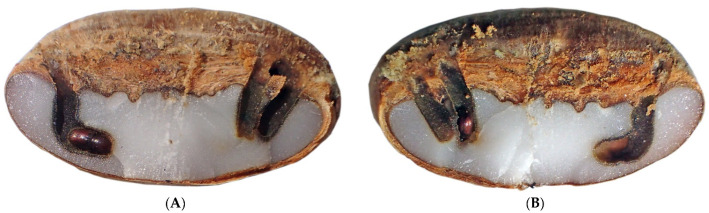
Triple penetration of a *Phoenix canariensis* seed with specimens killed due to cold exposure. (**A**) Left side of split seed; (**B**) right side.

Instances where a seed was penetrated by more than one beetle was a common pattern in experiments where bulk samples of seeds were exposed to multiple *C. dactyliperda* at the same time (Table 4). The penetration holes can be at opposite sides of the dorsal groove, but more often than not they are close to each other at the same location (Figure 3, Figure 4 and Figure 5). In total, 27.4% of the penetrations of seeds in bulk samples were multiples, ranging from two penetrations to five. In all but four cases, the multiple penetrations were located in the dorsal groove. Among germinated seeds, the percentage of double penetrations was less (19.4%). All multiple penetrations of seeds with open opercula also occurred from the dorsal groove.

### 3.2. Nature of Penetration

*Coccotrypes dactyliperda* readily penetrated *P. canariensis* seeds. In all bar one instance the seeds extracted from fresh *P. canariensis* drupes had been penetrated during the period between placement and the first subsequent observation (8 h artificial night or 16 h artificial day) [26]. On one occasion, two sample sets (prepared for a benchtop experiment) had already been penetrated after 2¾ hours.

The acceptance of germinated seeds depended on the moisture content of the seed. While all fresh seeds and those still moist from the germination experiment were readily accepted, the seeds that had been allowed to dry out for seven days were initially largely rejected. At the time of observation, almost 24 h after commencement of the experiment, 13 of the 30 beetles placed with black and dried out seeds (2017 season) were still found to be crawling about in the sample container. Among the dried brown seeds (2018 season), 12 of 30 beetles were found crawling. When the seeds were returned to the sample container, and the container vigorously shaken, another 11 beetles fell out, indicating that in each case the penetration had not progressed far.

In total, 6 h and 53 min of footage was collected recording four different penetration events. These were penetrations of *Phoenix canariensis* (excreta experiment, 6:14:03 h recorded, 2:21:18 h analyzed), *Corylus avellana* (sample AG6; 20:57 min), *Washingtonia robusta* (sample AH13; 14:28 min), and a blank for a Tagua button (sample U7; 3:26 min) (Table 5).

The beetles chewed into the epicarp in a circular fashion by regularly repositioning themselves in approximately one-eighth to one-quarter turns (Table 6 and Table 7; Figure S21). This repositioning can be clockwise or counter-clockwise, with changes in direction in the same gnawing sequence. The four recorded penetration events demonstrate the behavior of the beetles, but also highlight differences based on the nature of the seeds to be tunneled (Tables S3–S10).

## 4. Discussion

*Coccotrypes dactyliperda* tend to penetrate *P. canariensis* seeds in a systematic fashion with little variation. This appears to be driven by the nature of the seed’s surface. The data show an overwhelming preference for penetration at the dorsal side and in particular the dorsal groove (Table 2). This is likely to have a biomechanical reason. In order to penetrate, the date stone beetle needs to be able to push its mandibles into the epicarp of the seed. As this requires some force, the beetle needs to find sufficient traction. Biomechanically, the main thrust of the tunneling beetle is generated by the hind legs. Interpreting the footage, it appears that the mid legs function as a pivot, while the fore legs enable lateral control. We are less well informed once the beetle has created a full tunnel cavity, as observations are much more difficult. It would appear that inside the tunnel the pair of mid legs act as wall anchors.

The convex shape of the recto side of *P. canariensis* seeds does not provide any traction, whereas the dorsal groove side allows the beetle to stem against one or both sides while penetrating. There is a preference to penetrate the proximal end of the dorsal groove (Figure 2, sector ‘l’) rather than the end of the groove at the apex of the drupe. This is likely also a manifestation of traction, as the groove at the proximal end is more irregular. The beetles made use of existing access points, such as the open opercula of germinated seeds (samples #6–8), but not overwhelmingly so.

By and large, the same holds true for *Phoenix dactylifera* seeds, even though their surface tends to be rougher, providing more traction even on the ventral side. The dorsal groove of commercial date palm seeds is not as deep but more irregular and pitted, providing the beetle with increased opportunities for starting a tunnel (Figures S12 and S13).

In his experiments with date stone beetles penetrating buttons made from Tagua nuts, Herfs [6,7] observed the lack of traction on the smooth surfaces and noted that the beetles tended to penetrate the buttons from the thread holes. The same was observed by the present author among polished and hard (dried out) tagua (corozo) buttons used in the food choices experiment [26]. In these instances, the beetles penetrated from inside the holes in the button (Figures S14–S16) or used existing cracks in the fabric (Figure S17). Once the buttons had been rehydrated by prolonged soaking in water, some penetration occurred at the edges of the buttons where minor ornamental grooving provided the beetles with anchor points for their hind legs. 

Similarly, the majority of hazelnuts that were offered to *C. dactyliperda* showed successful penetration as well as incomplete penetration events primarily on the rough surface of the proximal end (Figures S18, S19 and S21), or on its very edge, tunneling into the sides (Figure S20). Where traction opportunities existed, penetration holes tended to be round with a smoothly convex bottom until full penetration was achieved (Figure S19). Where traction for the mid and/or hind legs was insufficient, penetration holes were more diffuse (Figure S20).

### 4.1. Tunneling and Gallery Building Process

The tunneling proceeds in a systematic fashion. In all cases, the tunnel proceeded straight down into the albumen (Figure 5) and then made a sharp L-shaped turn some 0.5 mm before it reached the epicarp (Figure S11). No cases were observed during the tunneling study or in other experiments where a beetle tunneled straight through to the other side.

Similar L-shaped tunnels were found in all other seeds, including those of *Phoenix dactylifera*. The tunnel section running parallel to the outer surface of the seed was gradually extended into a gallery that was continually enlarged by larvae and adult females. For much of the time a 0.5 to 0.8 mm thick section of albumen remained until late in the piece, when most albumen had been consumed. At that time the seed was either abandoned, or the albumen eaten out to the very edge of the epicarp (Figure S10). No cases were observed during the tunneling study and other experiments where a beetle had tunneled straight through to the other side. Both the position of the dog leg of the L-shaped tunnel and the fact beetles do not tunnel straight through the seed suggest that the albumen of the seed must exhibit some chemical changes that give gustatory cues to *C. dactyliperda* that the edge of the epicarp is close. As the seeds used in the majority of the experiments were freshly de-fleshed, an oxidization of the seed’s periphery and the development of a ‘patina’ can be excluded. 

None of the seeds that were split open for examination show the initial tunnel turning towards the proximal end of the seed. The dog-leg section is always pointing towards the center. At this point it is unclear whether gustatory cues are involved as it is noteworthy that the remaining albumen between the turning point of the tunnel and the proximal end of the seed is thicker than the albumen that remains between the dogleg and the edge of the seed.

Where multiple beetle specimens tunneled into the same seed, tunnels can clearly be seen very close to each other (Figure 5). It is unclear to what extent the different females will tolerate each other once the two (or more) tunnels (or galleries) merge. We know, for example, that if a female is removed, and there is no female offspring to take her place, the brood chamber may be occupied by another female. In such an instance the new female tends to clear the chamber of its precedecessor’s eggs and commence its own breeding [6,7].

**Figure 6:**
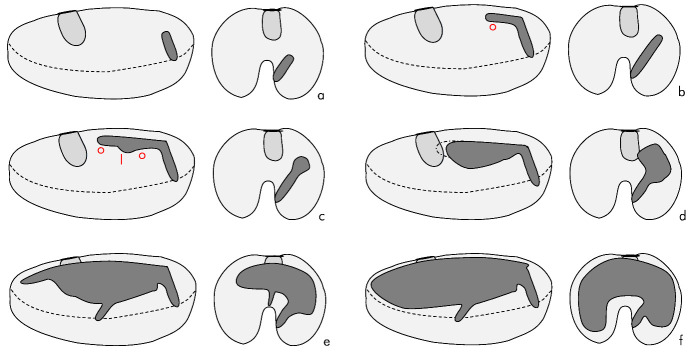
Schematic progressive development of *Coccotrypes dactyliperda* galleries inside a *Phoenix canariensis* seed. Shown are longitudinal and traversal cross-sections. l—larvae, o—oviposition. The approximate position of the embryo is indicated by a medium-grey shading.

Based on observations of seeds cut open at various stages of development, a schematic model of the development of *C. dactyliperda* galleries can be developed (Figure 6). The initial tunnel progresses straight but at an angle, into the albumen (a). The angle is variable and seems to be determined by the nature of the initial foothold that the beetle can attain. Some 0.5 to 0.8 mm from the margin of the seed, the tunnel makes an angular, ‘dog-leg’ turn towards the center of the seed (b). At this point oviposition occurs. The tunnel is then extended both in length as well as in depth, creating a small brood gallery (c). This gallery is extended both in width and in length (d). At this point the gallery may reach the seed’s embryo. The galleries are then expanded by both the founding female as well as her daughters, some of which may leave the seed via a new exit hole (e). Eventually most if not all of the albumen will be consumed (f).

None of the seeds that were split open for examination show damage to the embryo. Even if the embryo is avoided by the female, the continual expansion of the galleries will reduce any albumen available to the emerging cotyledonary petiole. In numerous cases the seed never germinated, with the residual embryo remaining in a shriveled state but with all adhering albumen cleanly removed. Predation of seeds by *C. dactyliperda* may not prevent germination but will certainly severely compromise seedling survival and success.

### 4.2. Patterns of Turning

The hardness of the penetrated material determines the amount of debris that is generated in a set time and therefore influences the time intervals between debris removal. A date stone beetle penetrating into a *Washingtonia robusta* seed, for example, tunneled for variable intervals (4:01, 2:10, 0:45, 2:36, 1:25 min) before ejecting debris, whereas a beetle tunneling into the epicarp of a hazelnut (*Corylus avellana*) gnawed for a total of 7:30 and 7:19 min before ejecting debris (see Tables S1 and S3 for details).

In no instance did a beetle complete a full 360° rotation during tunneling. In the majority of cases the beetle carried out only one turn before switching directions (Table 8). A full circle was recorded only during a debris removal sequence (excreta) when the beetle cleaned out the NW sector for 58 s and then turned clockwise. The majority of the stops in each octant was less than 15 s, with the exception of NE, where the beetle spent 2 min 17 s clearing out debris, and the W octant, where the beetle spent 38 s. The beetle continued to complete the full circle and then back tracked counter-clockwise, cleaning the next two octants again before recommencing tunneling (Table S9).

### 4.3. Exit Holes

While none of the experiments reported here were continued to the state that the first generation of beetles emerged, some observations were made on seeds used for food choice experiments reported elsewhere [26]. Observations showed that the *P. canariensis* seeds exhibited multiple emergence holes, all of which were located in the central dorsal groove (Figure S21). The number of exit holes varied. One of the seeds used for food choice examinations (sample AB5) had eleven exit holes. The founding female in that seed produced 93 offspring (incl. larvae and pupae) over a 45-day period (when the experiment was terminated). The fact that no exit holes were noted on the ventral side, or the bulging margins of the dorsal side, reinforces the presence of gustatory cues of some description that prevent *C. dactyliperda* from tunneling through the epicarp from anywhere but the central dorsal groove.

## 5. Conclusions

The study has shown that the tunneling of *C. dactyliperda* into seeds does not occur at random but follows specific patterns which seem to be governed by biomechanical constraints, in particular the ability of the beetle to gain traction. The tunneling and development of the brood gallery inside the seed are governed by gustatory cues which prevent the beetle from tunneling through to the other side. The nature of these gustatory cues remains unclear at this point and awaits future examination.

A further line of enquiry needs to consider whether or not there are patterns in the perforation of fresh drupes with the pericarp adhering. If traction is the primary driver for the location of the tunnel, then the seeds of penetrated full drupes should show a more random distribution of penetration hole locations.

## Figures and Tables

**Table 1:** Parameters of penetration samples.

Sample	Seeds	Exposure	Beetles	Number of Beetles Used	Seeds with Single Penetrations
#1	seed from fresh drupes	multiple	multiple	210	134
#2	seed from fresh drupes	multiple	Multiple	150	100
#3	seed, mixed	single, gridded	multiple	>90	90
#4	seed, dry	single, gridded	multiple	>56	56
#5	seed, mixed	single	single	82	82
#6	germinated seed, dry	multiple	multiple	30	30
#7	germinated seed, dry	multiple	multiple	30	15
#8	germinated seed, black	multiple	multiple	30	23

**Table 2:** Frequency (in %) of the location of the initial entry hole(s) in *Phoenix canariensis* seeds by observation and random generation, with testing for the probability that location l is preferred over others. n.s. = not significant. See Figure 2 for location codes.

	Exposure Type	Distribution		Bulk		Single			Bulk		
	Seed State	of Probability		Fresh		Fresh			Open Operculum		
	Sample	Equal	Random	#1	#2	#3	#4	#5	#6	#7	#8
Side	Sector										
Ventral	a	5.26	4				3.6				
	b	5.26	5				5.4	1.2			
	c	5.26	3				3.6				
	d	5.26	4								
	e	5.26	5	0.8		2.2	1.8	1.2			
	f	5.26	7								
	g	5.26	2								
	h	5.26	7				3.6				
	i	5.26	5		1.0		1.8				
	Operculum	5.26	6						33.33	13.33	30.43
Dorsal	k	5.26	5								
	l	5.26	8	74.6	59.0	62.0	51.8	37.8	23.33	73.33	39.13
	m	5.26	6	1.5			1.8				
	n	5.26	10			1.1	1.8				
	o	5.26	3	9.0	15.0	10.9	5.4	26.8	26.67	6.67	26.09
	p	5.26	6		3.0	1.1		1.2			
	q	5.26	7								
	r	5.26	3	13.4	22.0	22.8	19.6	30.5	16.67	6.67	4.35
	s	5.26	4					1.2			
n		100	100	134	100	90	56	82	30	15	23
χ^2^				110.649	65.767	69.435	44.954	29.643		49.193	
df				1	1	1	1	1		1	
*p*		n.s.	n.s.	<0.01	<0.01	<0.01	<0.01	<0.05	n.s	<0.01	n.s.

**Table 3:** Frequency (in %) of the location of the initial entry hole(s) in *Phoenix canariensis* seeds and testing for the probability that the rough dorsal groove is preferred over the smooth surfaces.

	Bulk		Single			Bulk		
	Fresh		Fresh			Open Operculum		
Side	#1	#2	#3	#4	#5	#6	#7	#8
Groove	96.6	96.0	95.7	76.8	95.1	66.7	86.7	69.6
Smooth Surface	3.0	4.0	4.4	23.2	4.9	-	-	-
Operculum	0.4	-	-	-	-	33.3	13.3	30.4
n	263	100	90	56	82	30	15	23
χ^2^	295.237	163.735	154.244	85.296	145.579	55.062	64.013	53.540
df	1	1	1	1	1	1	1	1
*p*	<0.001	<0.001	<0.001	<0.001	<0.001	<0.001	<0.001	<0.001

**Table 4:** Frequency (absolute number) of multiple penetrations of single *Phoenix canariensis* seeds. See Figure 2 for location codes.

Penetrations	Combination	Fresh Drupes Exp. #1	GERMINATED Exp. #6–#8
5	l (× 3), o (×2)	1	
4	k, l, o, r	1	
	l (× 2), o (× 2)	1	
	l, o (× 2), r	1	
3	l (× 3)	3	
	l (× 2), r	4	
	l (× 2), o	3	
	l (× 2), p	3	
	l, r (× 2)	2	
	l, o (× 2)		1
	l, o, r	1	
2	l, r	18	
	l (× 2)	19	3
	r (× 2)	5	1
	o (× 2)	1	
	o, l	7	1
	o, r	2	2
	operculum, l		3
	operculum, o		2
Total Sample (*n*)		263	68
Percent multiples		27.4	19.1

**Table 5:** Documented activities (hh:mm:ss).

Sample	Material	Hardness	Tunneling	Debris Removal	Pause	Wriggles Legs	Total
AG6	*Corylus avellana*	very hard	00:13:53	00:01:20	00:05:42	00:00:02	00:20:57
AH13	*Washingtonia robusta*	soft	00:13:14	00:01:14			00:14:28
U7	Tagua button blank	hard	00:03:26				00:03:26
Excreta	*Phoenix canariensis*	soft	01:39:38	00:41:40			02:21:18

**Table 6:** Proportions of directional turns (in %)—number of turns.

Sample	Counter-Clockwise	Clockwise	No Change	*n*
AG6	31.8	47.7	20.5	44
AH13	78.6	17.9	3.6	28
U7	16.7	79.2	4.2	24
Excreta	50.0	50.0	0.0	70
Total	45.2	48.2	6.6	166

**Table 7:** Proportions of directional turns (in %)—duration (hh:mm:ss).

Sample	Counter-Clockwise	Clockwise	No Change	Total
AG6	42.3	31.4	26.3	00:14:58
AH13	86.3	9.1	4.7	00:13:14
U7	7.3	88.3	4.4	00:03:26
Excreta	54.1	45.9	0.0	1:33:18
Total	54.8	41.4	3.8	2:04:56

**Table 8:** Frequency of consecutive turns in the same direction. Excreta sample.

	Consecutive Turns						
	1	2	3	4	5	6	*n*
clockwise	9		2	1			12
counter-clockwise	8	2	1			1	12
Total	17	2	3	1	1	1	24

## Supplementary Materials

The following are available online at https://www.mdpi.com/article/10.3390/insects13010010/s1, Figure S1. Sample preparation for the penetration experiment. Right; drupes as collected; left: extracted seeds, bottom: discarded pericarp’ center: 200 mL contained with 10 beetles. The dental rolls (bottom left) were moistened to provide a higher level of humidity. Figure S2. *Coccotrypes dactyliperda* exploring fresh drupes of Phoenix canariensis. Figure S3. Multiple perforations of *Phoenix canariensis* seeds. Figure S4. Triple perforation of a *Phoenix canariensis* seed. Details of Figure S3a. Figure S5. Triple perforation of a *Phoenix canariensis* seed. Details of Figure S3b. Note the small, attempted hole above the main hole (b). Figure S6. Dual perforations of *Phoenix canariensis* seeds. Details of Figure S3c (left); Figure S3d (right). Figure S7. Dual perforations of *Phoenix canariensis* seeds. Details of Figure S3e (left); Figure S3f (right). Figure S8. Dual perforations of *Phoenix canariensis* seeds. Details of Figure S3g (left); Figure S3h (right). Figure S9. Dual perforations of *Phoenix canariensis* seeds. Details of Figure S3i. Figure S10. Largely eaten-out *Phoenix canariensis* seed (AB5). Figure S11. *Phoenix dactylifera* seed and galleries (O3). Figure S12. Tunnelling into a vintage tagua button (Q7). Figure S13. Tunnelling into a vintage tagua button (Q4). Figure S14. Tunnelling into a vintage tagua button (Q12). Figure S15. Penetration attempt of a hazelnut (AG5). Figure S16. Abandoned penetration attempts of a hazelnut (AG1). Figure S17. Abandoned penetration attempt of a hazelnut (AG5). Note the convex nature of the base of the hole. Figure S18. Abandoned penetration attempt of a hazelnut. (AG6, repeat nº 1). Stills from a video. Figure S19. Tunnelling sequence of *Washingtonia robusta* (AH13).Figure S20. Tunnelling into a button blank (U7). Figure S21. Multiple emergence (exit) holes in *Phoenix canariensis* seeds. Table S1. Frequency (%) of the location of the initial entry hole(s) in *Phoenix canariensis* seeds and the probability that location l is preferred over others: Experiment #1. See Figure 2 (in main text) for location codes. Table S2. Frequency (%) of the location of the initial entry hole(s) in *Phoenix canariensis* seeds and the probability that location l is preferred over others: Experiment #2. See Figure 2 (in main text) for location codes. Table S3. Tunnelling action, hard epicarp (hazelnut, sample AG6). Times taken from video. For stills see Figure S18. Abbreviations: CCW–counter-clockwise; CW–clockwise; DR–debris removal; G–gnawing; P–pause. Table S4. Orientation of tunnelling action, hard epicarp (hazelnut, sample AG6). For detailed data see Table S3. Table S5. Tunnelling action, albumen (*Washingtonia robusta*, sample AH13). Times taken from video. For stills see Figure S19. Abbreviations: CCW–counter-clockwise; CW–clockwise; DR–debris removal; G–gnawing. Table S6. Orientation of tunnelling action, albumen (*Washingtonia robusta*, sample AH13). For detailed data see Table S5. Table S7. Tunnelling action, expanding tunnel (tagua button blank, sample U7). Times taken from video. For stills see Figure S20. Abbreviations: CCW–counter-clockwise; CW–clockwise; G–gnawing. Table S8. Orientation of tunnelling action, albumen (tagua button blank, sample U7). For detailed data see Table S7. Table S9. Tunnelling action (*Phoenix canariensis*). Times taken from video. For stills see Figure S20. Abbreviations see Table S3. Table S10. Orientation of tunnelling action, hard epicarp. For detailed data see Table S9.
