# Supplementary material for: Patterns of Seed Penetration by the Date Stone Beetle Coccotrypes dactyliperda (Coleoptera, Curculionidae, Scolytinae)
Source: Insects. 2021 Dec 22;13(1):10. doi: 10.3390/insects13010010 (PMC8781270; doi:10.3390/insects13010010)
Supplement: Supplementary file 1 [file insects-13-00010-s001.zip › insects-1469956-supplementary.pdf]

insects

Supplementary Material

Patterns of seed penetration by the date stone beetle *Coccotrypes dactyliperda* (Coleoptera, Curculionidae, Scolytinae)

Dirk H.R. Spennemann [1,*]

This document presents supplementary data tables as well as photographic documentation of penetration actions in support of the following paper:
Spennemann, Dirk H.R. (2021). Patterns of seed penetration by the date stone beetle *Coccotrypes dactyliperda* (Coleoptera, Curculionidae, Scolytinae). Insects

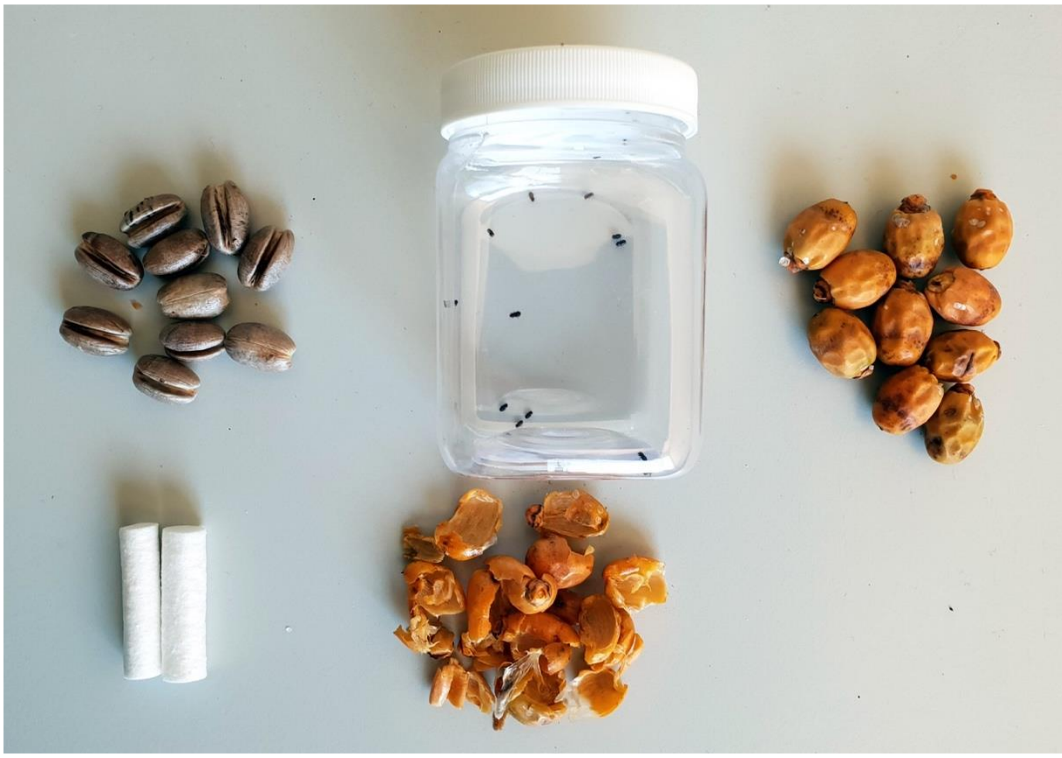

Figure S1. Sample preparation for the penetration experiment. Right; drupes as collected; left: extracted seeds, bottom: discarded pericarp' center: 200ml contained with 10 beetles. The dental rolls (bottom left) were moistened to provide a higher level of humidity.

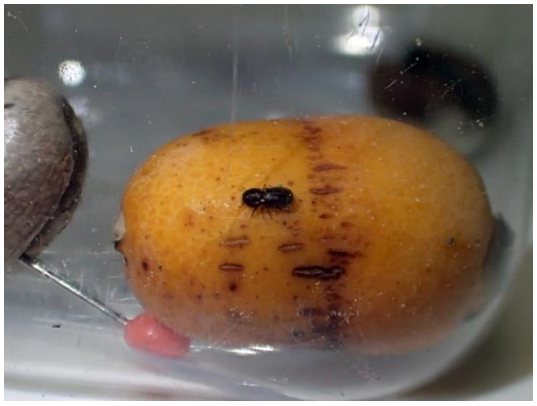

Experiment D3

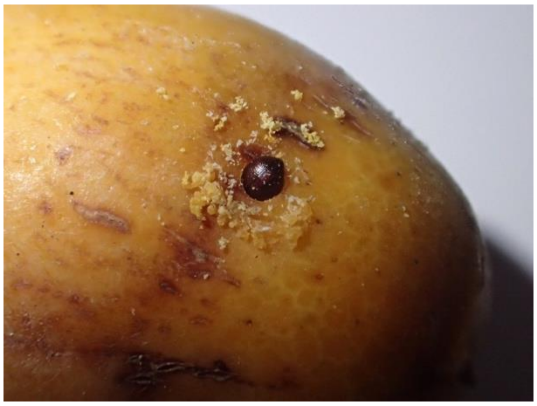

Experiment D4

Figure S2. Cooctryoes dactyliperda exploring fresh drupes of Phoenix canariensis

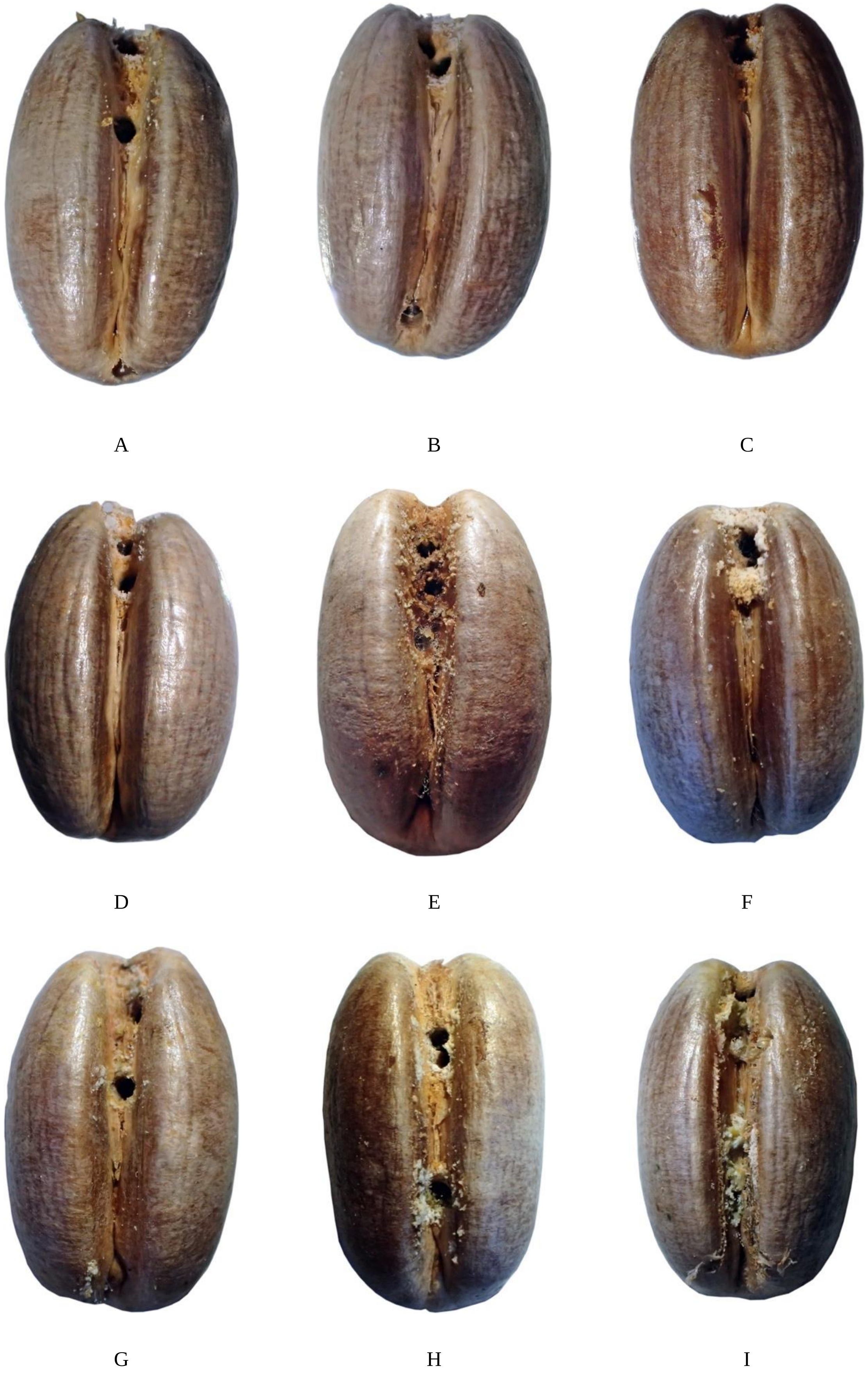

Figure S3. Multiple perforations of Phoenix canariensis seeds

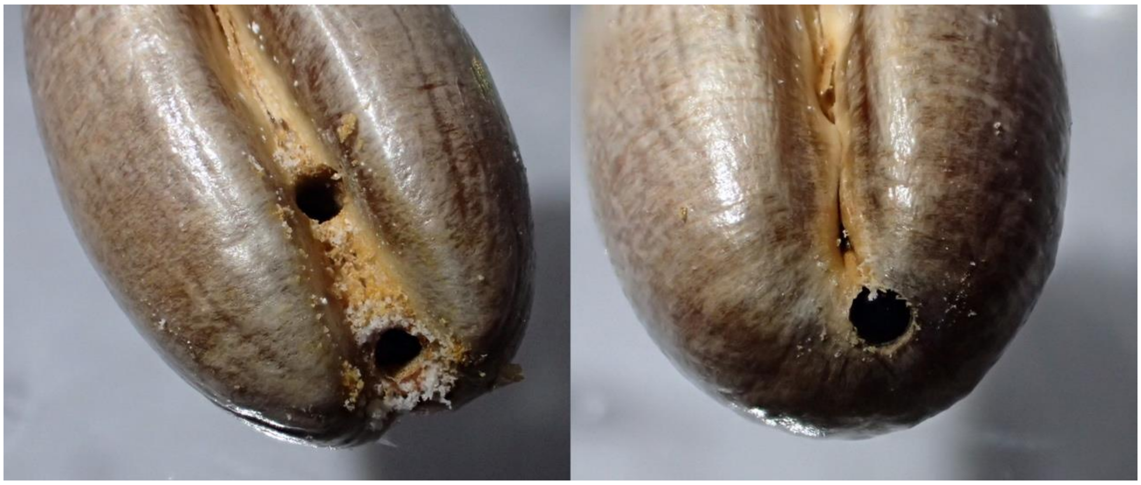

a b

Figure S4. Triple perforation of a Phoenix canariensis seed. Details of Figure S3a.

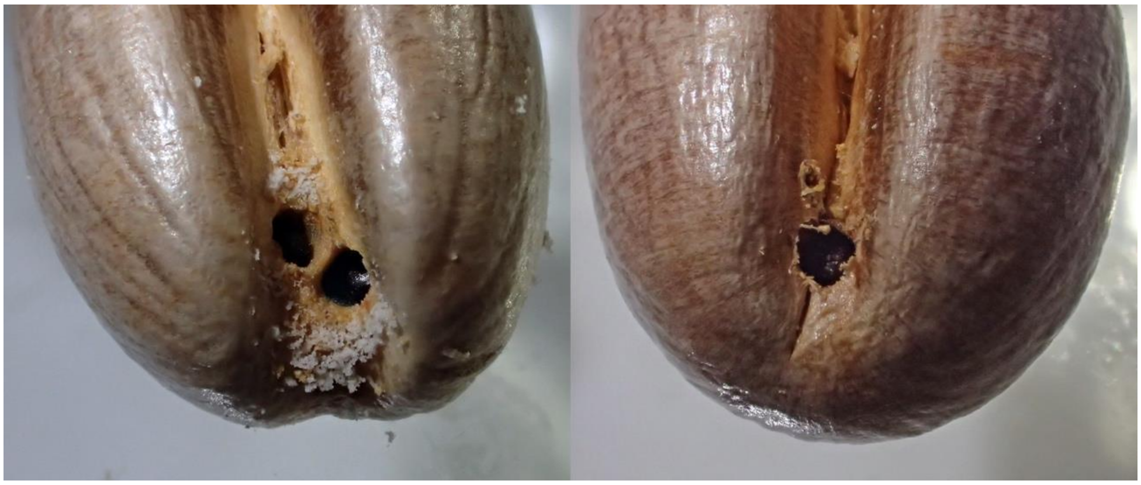

a b

Figure S5. Triple perforation of a Phoenix canariensis seed. Details of Figure S3b. Note the small, attempted hole above the main hole (b).

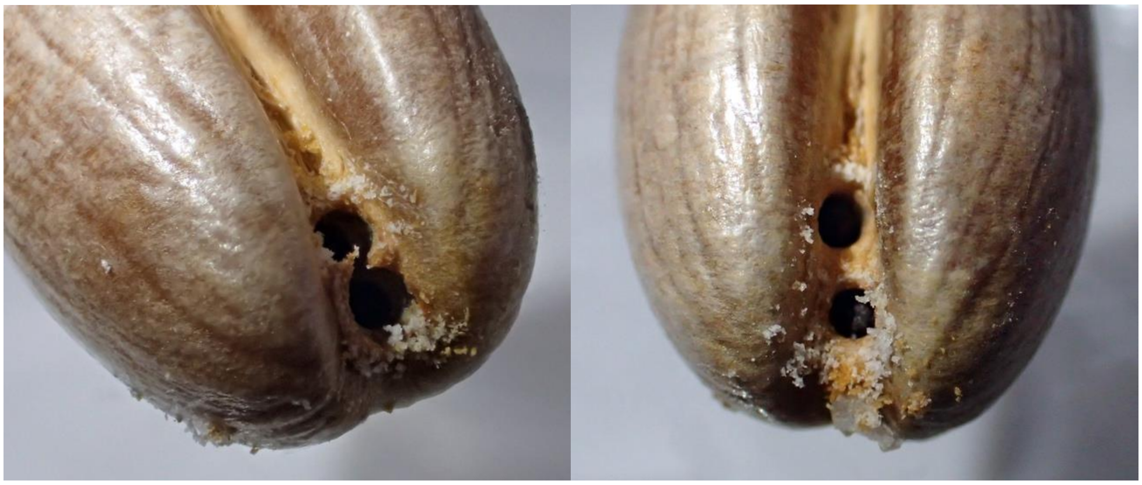

a b

Figure S6. Dual perforations of Phoenix canariensis seeds. Details of Figure S3c (left); Figure S3d (right)

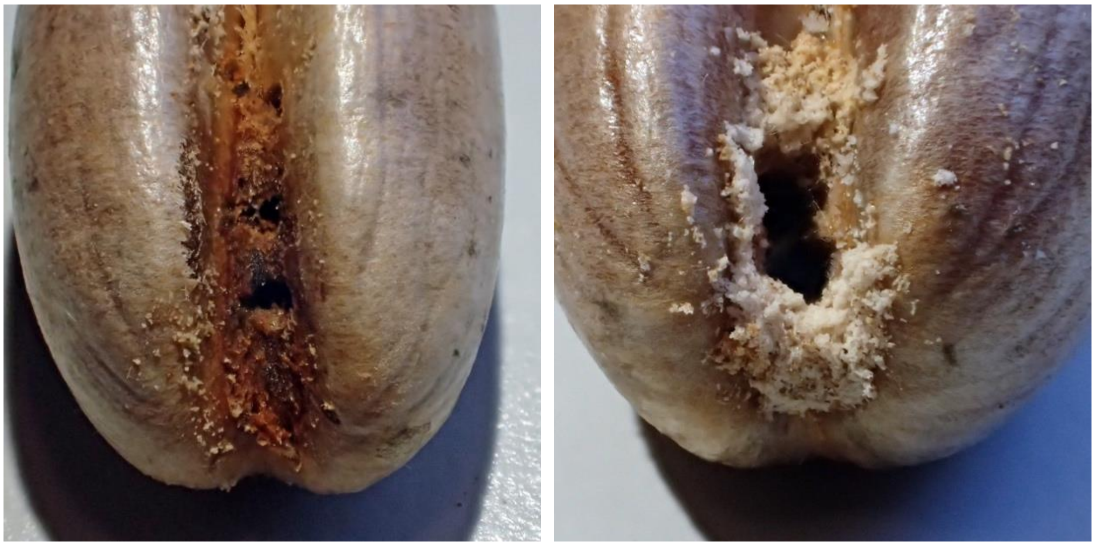

a b

Figure S7. Dual perforations of Phoenix canariensis seeds. Details of Figure S3e (left); Figure S3f (right)

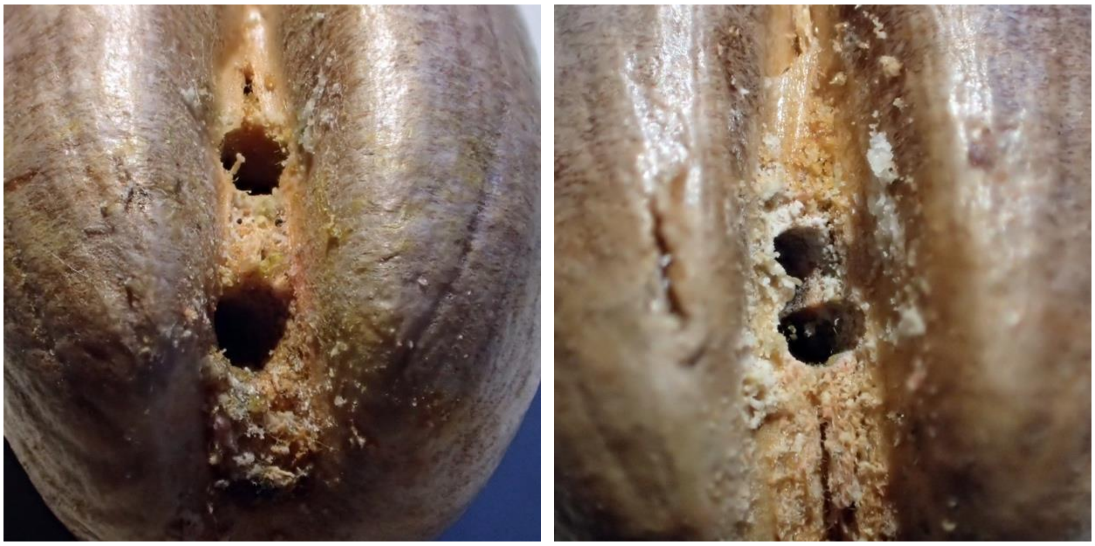

a b

Figure S8. Dual perforations of Phoenix canariensis seeds. Details of Figure S3g (left); Figure S3h (right)

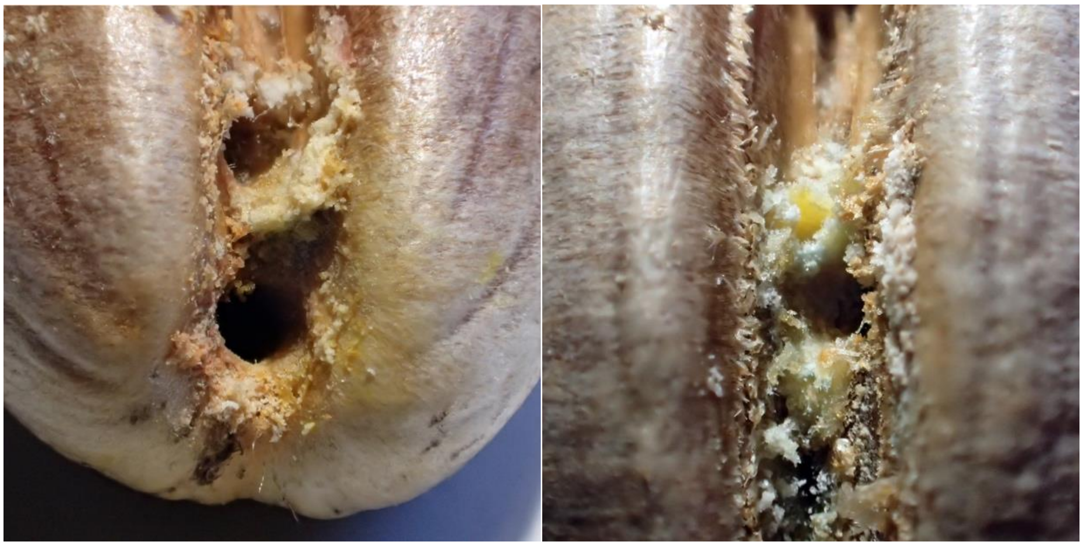

a b

Figure S9. Dual perforations of Phoenix canariensis seeds. Details of Figure S3i

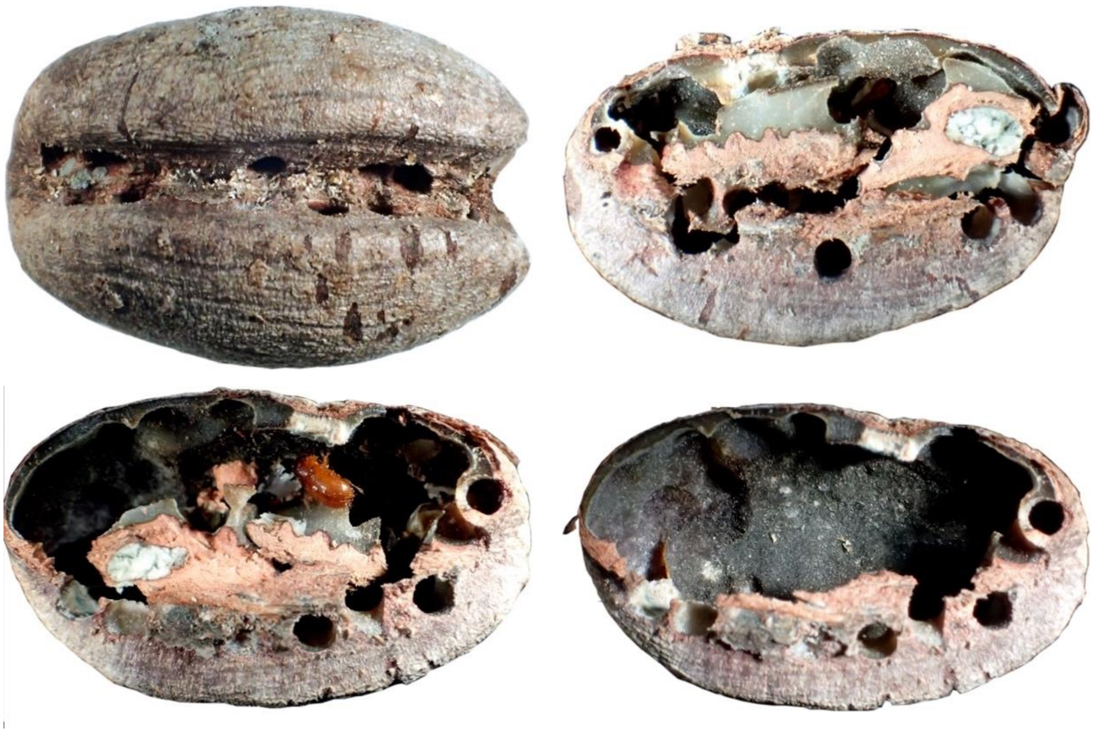

Figure S10. Largely eaten-out Phoenix canariensis seed (AB5)

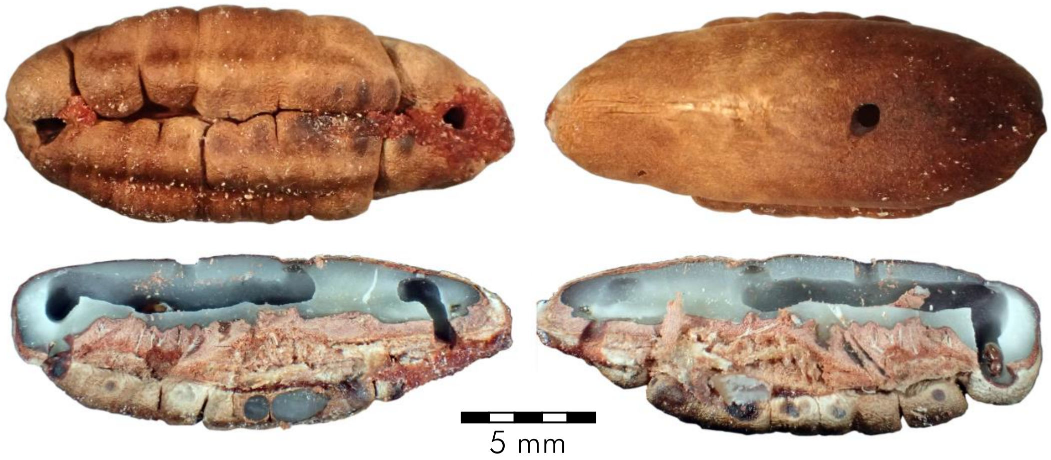

Figure S11. Phoenix dactylifera seed and galleries (O3)

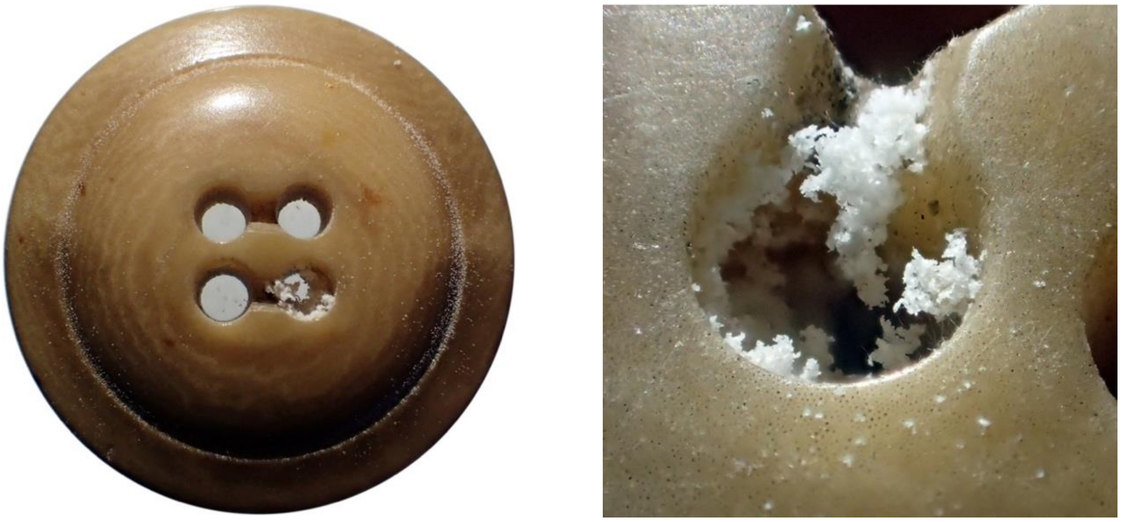

full button detail view

Figure S12. Tunnelling into a vintage tagua button (Q7)

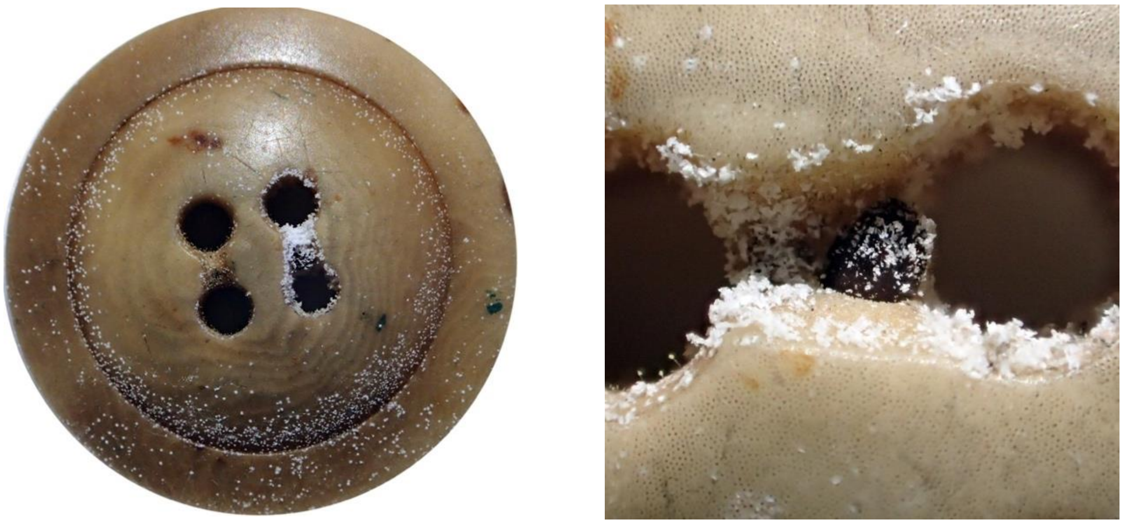

full button detail view

Figure S13. Tunnelling into a vintage tagua button (Q4)

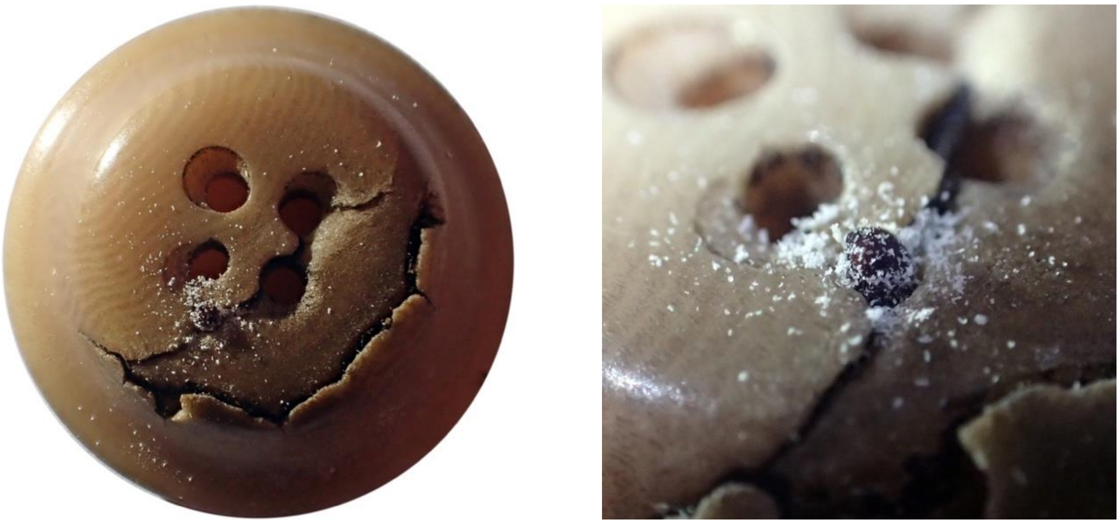

full button detail view

Figure S14. Tunnelling into a vintage tagua button (Q12)

Table S1. Frequency (%) of the location of the initial entry hole(s) in *Phoenix canariensis* seeds and the probability that location l is preferred over others: Experiment #1. See Figure 2 (in main text) for location codes.

Side	Sector	#1A	#1B	#1C	#1D	#1E	#1F	#1G	Total
recto	a								
	b								
	c								
	d								
	e				7.14				0.75
	f								
	g								
	h								
	i								
	operculum								
verso	k	7.14							
	l	64.29	75.00	80.00	71.43	62.50	75.86	100.00	74.63
	m		5.00			4.17			1.49
	n								
	o	7.14		5.00	14.29	16.67	13.79		8.96
	p								
	q								
	r	21.43	20.00	15.00	7.14	16.67	10.34		13.43
	s								
n		14	20	20	14	24	29	13	134
p		<0.01	<0.01	<0.01	<0.01	<0.01	<0.01	<0.01	<0.01

Table S2. Frequency (%) of the location of the initial entry hole(s) in *Phoenix canariensis* seeds and the probability that location l is preferred over others: Experiment #2. See Figure 2 (in main text) for location codes.

Side	Sector	#2A	#2B	#2C	#2D	#2E	#2F	#2G	#2H	#2I	#2J	Total
recto	a											
	b											
	c											
	d											
	e											
	f											
	g											
	h											
	i							10				1
	operculum											
verso	k											
	l	60	50	60	80	70	30	20	90	60	70	59
	m											
	n											
	o	20	20			20	30	30		20	10	15
	p			10	10	10						3
	q											
	r	20	30	30	10		40	40	10	20	20	22
	s											
n	n	10	10	10	10	10	10	10	10	10	10	100
p		<0.05			<0.01	<0.01			<0.01	<0.05	<0.01	<0.01

Table S3. Tunnelling action, hard epicarp (hazelnut, sample AG6). Times taken from video. For stills see Figure S18. Abbreviations: CCW–counter-clockwise; CW–clockwise; DR–debris removal; G–gnawing; P–pause.

Time	Direction	Rotation	Activity	Duration	Time	Direction	Rotation	Activity	Duration
0:00	N		G	0:06	8:15	SW	CCW	G	0:03
0:06	NW	CCW	G	0:05	8:18	S	CCW	G	0:15
0:11	W	CCW	G	0:34	8:33	SE	CCW	DR	1:05
0:45	NW	CW	G	0:05	9:38	SE		G	0:10
0:50	N	CW	G	0:04	9:48	E	CCW	G	0:07
0:54	NE	CW	G	0:02	9:55	E		P	2:27
0:56	E	CW	G	0:13	12:22	E		G	2:23
1:09	NE	CCW	G	1:05	14:45	E		G	0:24
2:14	N	CCW	G	0:50	15:09	NE	CCW	G	0:06
3:04	NW	CCW	G	0:16	15:15	N	CCW	G	0:20
3:20	W	CCW	G	0:45	15:35	NE	CW	G	0:05
4:05	W		P	0:26	15:40	E	CW	G	0:19
4:31	W		G	0:10	15:59	SE	CW	G	0:05
4:41	SW	CCW	G	0:34	16:04	S	CW	G	0:35
5:15	SW		P	0:37	16:39	S		P	0:34
5:52	SW		G	0:25	17:13	S		[1]	0:02
6:17	S	CCW	G	0:15	17:15	S		P	0:15
6:32	SW	CW	G	0:02	17:30	S		G	0:07
6:34	W	CW	G	0:07	17:37	S		P	1:23
6:41	NW	CW	G	0:04	19:00	S		G	0:06
6:45	N	CW	G	0:03	19:06	S		G	0:05
6:48	NE	CW	G	0:03	19:11	S		DR	0:15
6:51	E	CW	G	0:08	19:26	SW	CW	G	0:22
6:59	SE	CW	G	0:07	19:48	W	CW	G	0:31
7:06	S	CW	G	0:45	20:19	NW	CW	G	0:38
7:51	SW	CW	G	0:04	20:57	NW		[2]	
7:55	W	CW	G	0:20					

Notes: [1] beetle wriggled hind legs for two seconds; [2] extracts from hole and abandons the tunneling effort

Table S4. Orientation of tunnelling action, hard epicarp (hazelnut, sample AG6). For detailed data see Table S3.

Direction	Tunneling	Debris Removal	Pause	wriggles legs	Total
N	01:23				01:23
NE	01:21				01:21
E	03:34		02:27		06:01
SE	00:22	01:05			01:27
S	02:08	00:15	02:12	00:02	04:37
SW	01:30		00:37		02:07
W	02:27		00:26		02:53
NW	01:08				01:08
Total	13:53	01:20	05:42	00:02	20:57

Table S5. Tunnelling action, albumen (Washingtonia robusta, sample AH13). Times taken from video. For stills see Figure S19. Abbreviations: CCW–counter-clockwise; CW–clockwise; DR–debris removal; G–gnawing.

Time	Direction	Rotation	Activity	Duration	Time	Direction	Rotation	Activity	Duration
0:00	SE		G	0:37	6:56	E		DR	0:30
0:37	SE		DR	0:04	7:26	NE	CCW	G	0:10
0:41	E	CCW	G	0:22	7:36	E	CW	G	0:19
1:03	NE	CCW	G	0:29	7:55	NE	CW	G	0:16
1:32	N	CCW	G	0:50	8:11	NE		DR	0:05
2:22	NW	CCW	G	0:10	8:16	N	CCW	G	0:11
2:32	W	CCW	G	1:07	8:27	NE	CCW	G	0:57
3:39	SW	CCW	G	0:11	9:24	N	CCW	G	0:06
3:50	S	CCW	G	0:13	9:30	NW	CCW	G	1:22
4:03	SW	CW	G	0:04	10:52	NW		DR	0:13
4:07	W	CW	G	0:31	11:05	W	CCW	G	1:40
4:38	NW	CW	G	0:02	12:45	W		DR	0:03
4:40	W	CCW	G	0:02	12:48	SW	CCW	G	0:01
4:42	W		DR	0:04	12:49	S	CCW	G	0:04
4:46	SW	CCW	G	0:01	12:53	SE	CCW	G	1:20
4:47	S	CCW	G	0:02	14:13	SE		DR	0:15
4:49	SE	CCW	G	0:59	14:28	E	CCW	G	
5:48	E	CCW	G	1:08					

Table S6. Orientation of tunnelling action, albumen (Washingtonia robusta, sample AH13). . For detailed data see Table S5.

Direction	Tunneling	Debris Removal	Total
N	01:07		01:07
NE	01:52	00:05	01:57
E	01:49	00:30	02:19
SE	02:56	00:19	03:15
S	00:19		00:19
SW	00:17		00:17
W	03:20	00:07	03:27
NW	01:34	00:13	01:47
Total	13:14	01:14	14:28

Table S7. Tunnelling action, expanding tunnel (tagua button blank, sample U7). Times taken from video. For stills see Figure S20. Abbreviations: CCW–counter-clockwise; CW–clockwise; G–gnawing.

Time	Direction	Rotation	Activity	Duration	Time	Direction	Rotation	Activity	Duration
0:00	NW		G	0:09	02:29	NW	CCW	G	0:05
0:09	W	CCW	G	0:06	02:34	N	CW	G	0:07
0:15	NW	CW	G	0:05	02:41	NE	CW	G	0:03
00:20	N	CW	G	0:02	02:44	E	CW	G	0:04
00:22	NE	CW	G	0:02	02:48	SE	CW	G	0:02
00:24	E	CW	G	0:11	02:50	E	CW	G	0:13
00:35	SE	CW	G	0:08	03:03	SE	CW	G	0:03
00:43	S	CW	G	0:07	03:06	S	CW	G	0:05
00:50	SW	CW	G	0:14	03:11	SE	CCW	G	0:04
01:04	W	CW	G	0:05	03:15	S	CW	G	0:07
01:09	NW	CW	G	0:02	03:22	SW	CW	G	0:04
01:11	N	CW	G	1:18	03:26	S	CCW	G	

Table S8. Orientation of tunnelling action, albumen (tagua button blank, sample U7). For detailed data see Table S7.

Direction	Tunneling	Total
N	01:27	01:27
NE	00:05	00:05
E	00:28	00:28
SE	00:17	00:17
S	00:19	00:19
SW	00:18	00:18
W	00:11	00:11
NW	00:21	00:21
Total	03:26	03:26

Table S9. Tunnelling action (Phoenix canariensis). Times taken from video. For stills see Figure S20. Abbreviations see Table S3

Video	Time	Direction	Rotation	Activity	Duration
A01	00:00	N		Tunneling	03:07
A01	03:07	N		Debris Removal	02:46
A01	05:53	NE	CW	Tunneling	02:34
A01	08:27	N	CCW	Tunneling	05:26
A01	13:53	NW	CCW	Debris Removal	05:06
A01	18:59	N	CW	Tunneling	01:09
A01	20:08	NW	CCW	Tunneling	01:40
A01	21:48	NW			
New Video, gap 03:42					
A02	00:00	SE	CW	Tunneling	02:01
A02	02:01	E	CCW	Tunneling	00:13
A02	02:14	NE	CCW	Tunneling	01:28
A02	03:42	N	CCW	Tunneling	00:08
A02	03:50	NW	CCW	Debris Removal	00:32
A02	04:22	W	CCW	Tunneling	00:15
A02	04:37	SW	CCW	Tunneling	06:33
A02	11:10	S	CCW	Tunneling	00:12
A02	11:22	SW	CW	Tunneling	00:19
A02	11:41	W	CW	Tunneling	00:10
A02	11:51	NW	CW	Tunneling	01:46
A02	13:37	N	CW	Tunneling	
			New Video, gap 16:43		
A03	00:00	NE	CW	Tunneling	04:42
A03	04:42	NW	CCW	Debris Removal	02:49
A03	07:31	NW		Debris Removal	05:42
A03	13:13	W	CCW	Debris Removal	00:25
A03	13:38	NW	CW	Debris Removal	01:38
A03	15:16	N	CW	Debris Removal	00:24
A03	15:40	NE	CW	Tunneling	03:28
A03	19:08	E	CW	Debris Removal	00:17
A03	19:25	SE	CW	Debris Removal	00:52
A03	20:17	SE		Tunneling	
			New Video, gap 00:30		
A04	00:00	SE		Tunneling	02:09
A04	02:09	S	CW	Tunneling	03:07
A04	05:16	SE	CCW	Debris Removal	01:41
A04	06:57	E	CCW	Tunneling	01:34
A04	08:31	NE	CCW	Debris Removal	00:26
A04	08:57			Tunneling	04:05
A04	13:02	N	CCW	Debris Removal	00:23
A04	13:25	N		Debris Removal	01:52
A04	15:17	NW		Tunneling	05:42
A04	20:59				
New Video, gap 00:12					
A05	00:00	NW		Tunneling	00:24
A05	00:24	NW		Tunneling	
			New Video, gap 02:12		

Video	Time	Direction	Rotation	Activity	Duration
A06	00:00	SE		Debris Removal	00:26
A06	00:26	S	CW	Tunneling	02:53
A06	03:19	SW	CW	Tunneling	01:08
A06	04:27	W	CW	Tunneling	01:40
A06	06:07	NW	CW	Debris Removal	00:14
A06	06:21	NW		Tunneling	00:25
A06	06:46	W	CCW	Tunneling	01:18
A06	08:04	W		Debris Removal	00:15
A06	08:19	NW	CCW	Tunneling	02:16
A06	10:35	NW		Debris Removal	00:25
A06	11:00	N	CW	Tunneling	01:20
A06	12:20	N		Debris Removal	00:39
A06	12:59	NE	CW	Debris Removal	00:54
A06	13:53	N	CW	Tunneling	00:04

Table S9(ct'd). Tunnelling action (Phoenix canariensis). Times taken from video. For stills see Figure S23. Abbreviations see Table S3

Video	Time	Direction	Rotation	Activity	Duration
A06	13:57	NW	CCW	Tunneling	04:44
A06	18:41	NW		Debris Removal	00:33
A06	19:14	W	CCW	Debris Removal	00:09
A06	19:23	NW	CW	Tunneling	02:18
A06	21:41	NW		Tunneling	
		New Video, gap 01:02			
A07	00:00	NW		Debris Removal	00:30
A07	00:30	N		Debris Removal	00:12
A07	00:42	NE		Tunneling	01:08
A07	01:50	NE		Tunneling	00:05
A07	01:55	E		Tunneling	00:30
A07	02:25	SE		Tunneling	00:57
A07	03:22	SE		Debris Removal	01:01
A07	04:23	E		Tunneling	01:33
A07	05:56	NE		Debris Removal	00:17
A07	06:13	N		Tunneling	02:45
A07	08:58	N		Debris Removal	00:35
A07	09:33	NW		Debris Removal	00:12
A07	09:45	W		Debris Removal	00:27
A07	10:12	NW		Tunneling	03:09
A07	13:21	N		Debris Removal	00:21
A07	13:42	NW		Debris Removal	00:58
A07	14:40	N		Debris Removal	00:04
A07	14:44	NE		Debris Removal	02:17
A07	17:01	E		Debris Removal	00:05
A07	17:06	SE		Debris Removal	00:11
A07	17:17	S		Debris Removal	00:04
A07	17:21	SW		Debris Removal	00:15
A07	17:36	W		Debris Removal	00:38
A07	18:14	NW		Debris Removal	00:11
A07	18:25	W		Debris Removal	00:15
A07	18:40	SW		Debris Removal	00:05
A07	18:45	SW		Tunneling	02:00
A07	20:45	SW		Tunneling	
		New Video, gap 00:12			
A08	00:00	W		Tunneling	03:33
A08	03:33	W		Debris Removal	00:16
A08	03:49	W		Tunneling	02:55
A08	06:44	W		Debris Removal	02:39
A08	09:23	W		Tunneling	00:07
A08	09:30	SW		Debris Removal	00:23
A08	09:53	S		Tunneling	06:30
A08	16:23	SE		Debris Removal	00:03
A08	16:26	SE		Tunneling	00:03
A08	16:29	E		Tunneling	01:01
A08	17:30	SE		Tunneling	00:51
A08	18:21	SE		Debris Removal	00:21
A08	18:42	E		Tunneling	02:13
A08	20:55	NE		Debris Removal	00:52

Table S10. Orientation of tunnelling action, hard epicarp (¶¶¶). For detailed data see Table S9.

Row Labels	Tunneling	Debris Removal	Total
N	13:59	07:16	21:15
NE	17:30	04:46	22:16
E	07:04	00:22	07:26
SE	06:01	04:35	10:36
S	12:42	00:04	12:46
SW	10:00	00:43	10:43
W	09:58	05:04	15:02
NW	22:24	18:50	41:14
Total	1:39:38	41:40	2:21:18

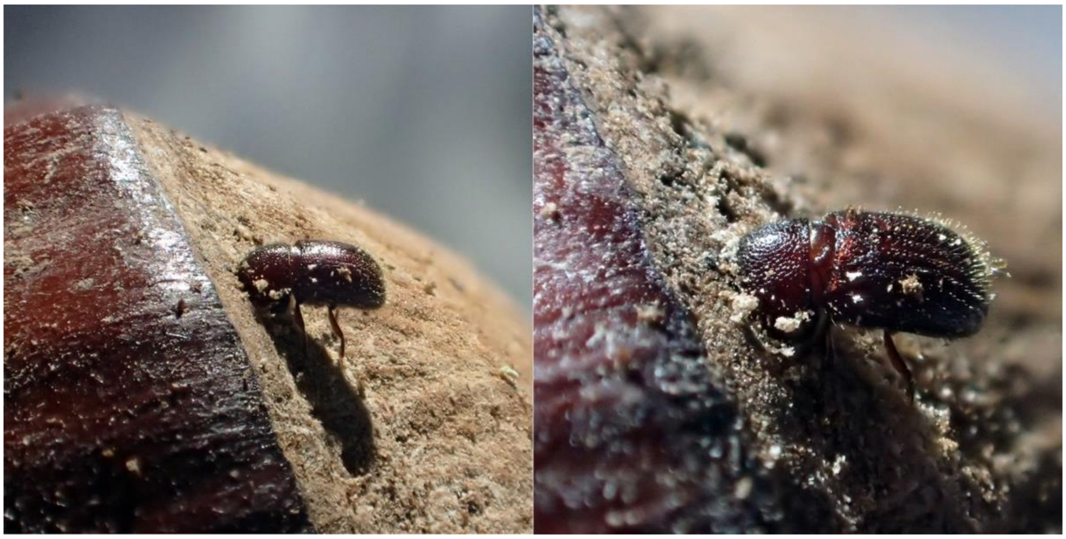

a b

Figure S15. Penetration attempt of a hazelnut (AG5).

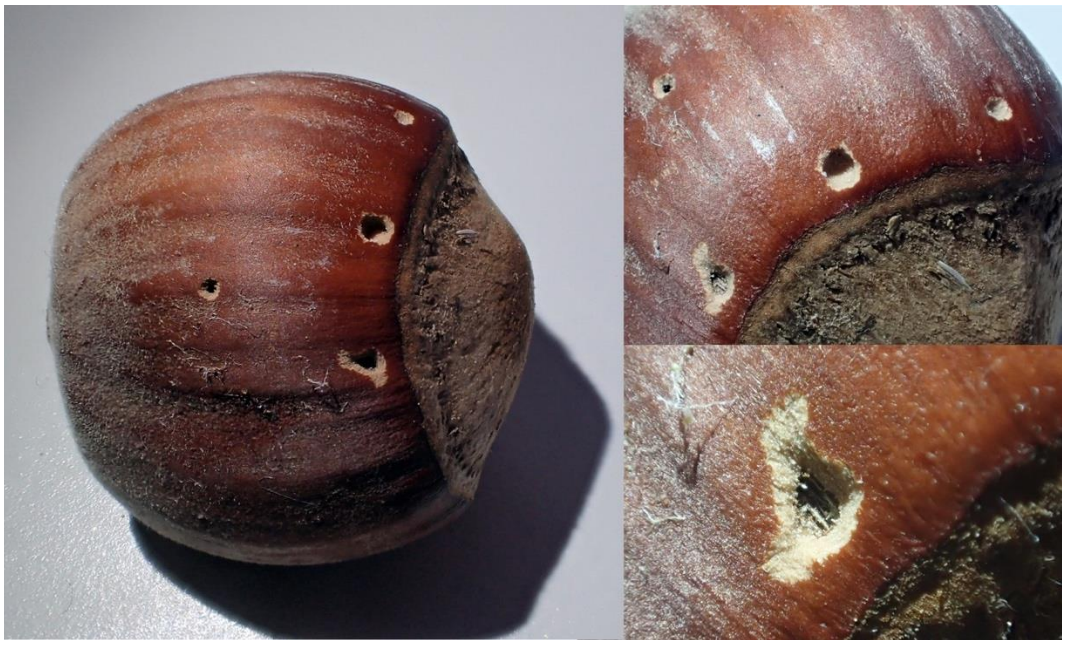

a b

Figure S16. Abandoned penetration attempts of a hazelnut (AG1).

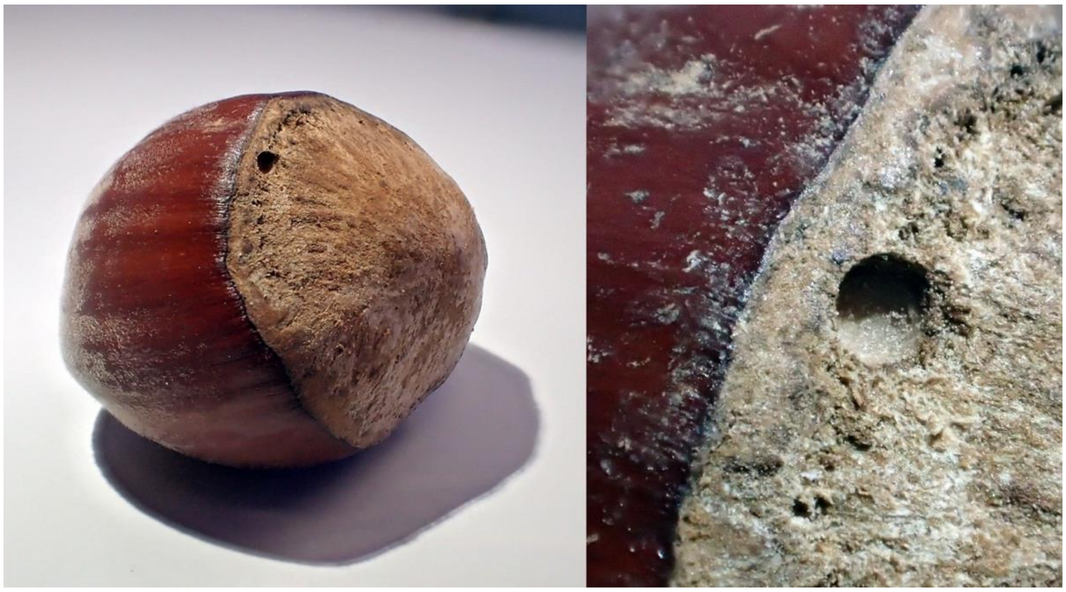

a b

Figure S17. Abandoned penetration attempt of a hazelnut (AG5). Note the convex nature of the base of the hole.

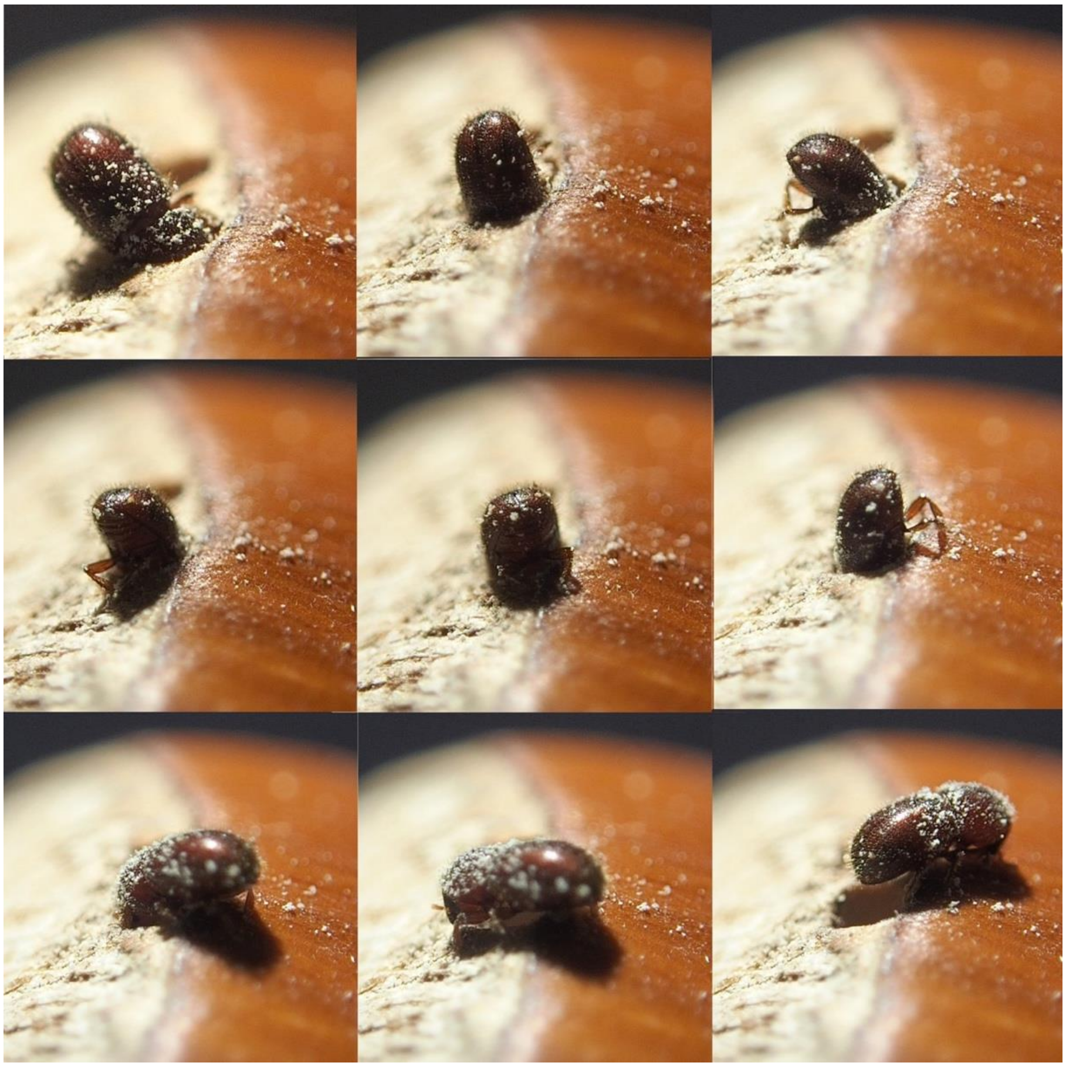

Figure S18. Abandoned penetration attempt of a hazelnut. (AG6, repeat nº 1). Stills from a video.

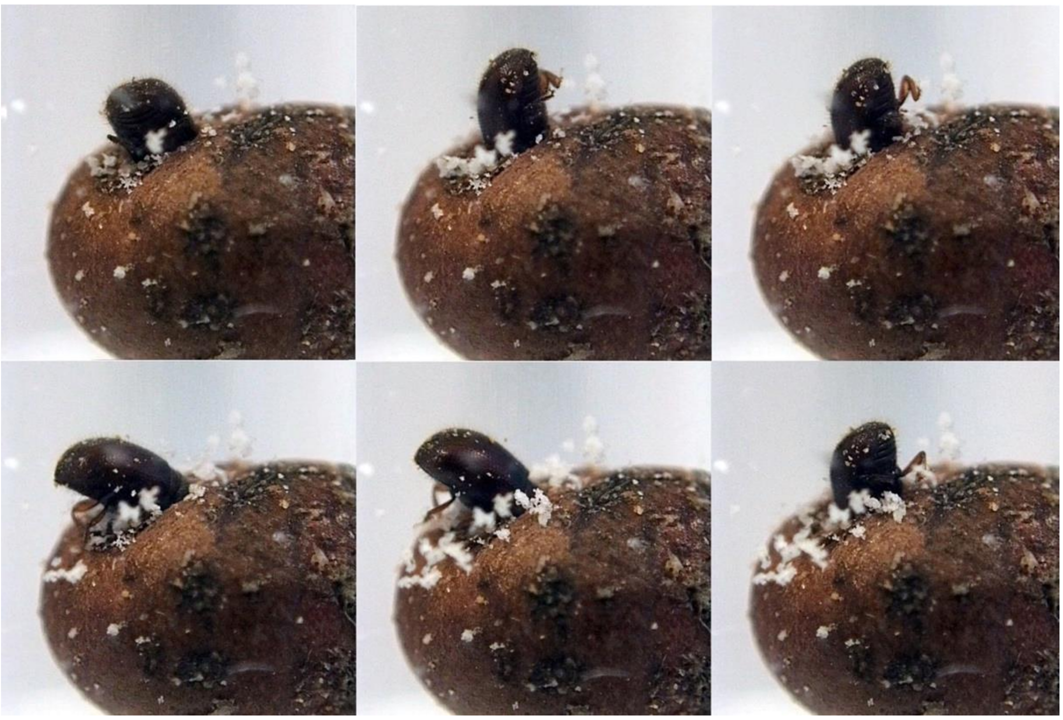

Figure S19. Tunnelling sequence of Washingtonia robusta (AH13).

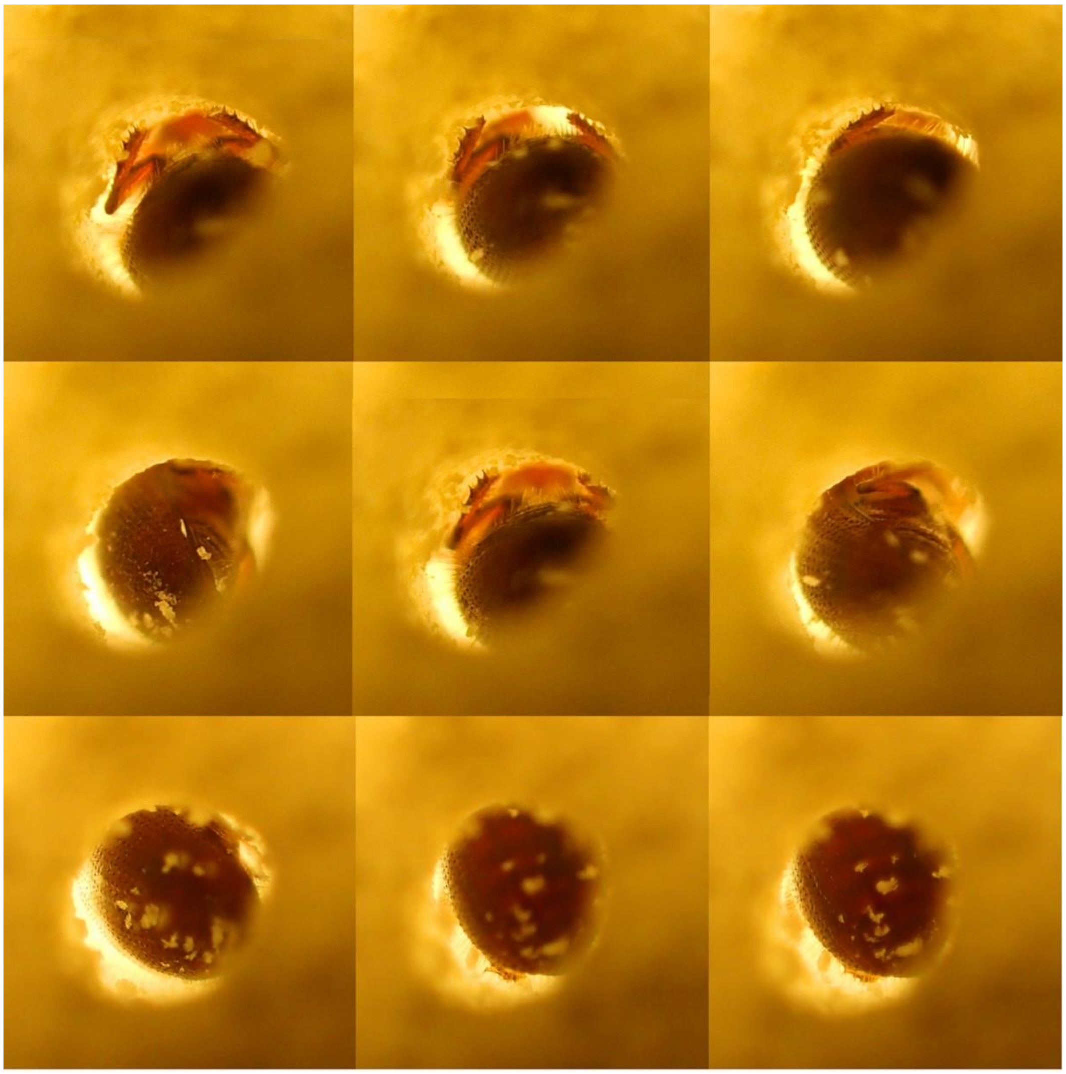

Figure S20. Tunnelling into a button blank (U7).

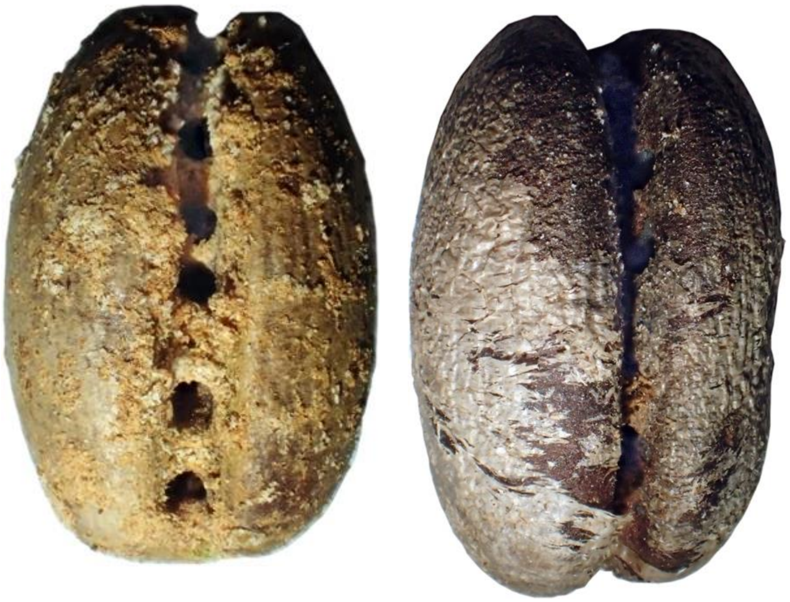

B1 C11

Figure S21. Multiple emergence (exit) holes in Phoenix canariensis seeds.
